# Experimental Verification of Isotropic and Anisotropic Anhysteretic Magnetization Models

**DOI:** 10.3390/ma12091549

**Published:** 2019-05-11

**Authors:** Michał Nowicki, Roman Szewczyk, Paweł Nowak

**Affiliations:** Warsaw University of Technology, Institute of Metrology and Biomedical Engineering, 02-495 Warsaw, Poland; szewczyk@mchtr.pw.edu.pl (R.S.); nowak@mchtr.pw.edu.pl (P.N.)

**Keywords:** magnetization model, anhysteretic magnetization curve, Mn-Zn ferrites, amorphous alloys

## Abstract

The anhysteretic magnetization curve is the key element of modeling magnetic hysteresis loops. Despite the fact that it is intensively exploited, known models of anhysteretic curve have not been verified experimentally. This paper presents the validation of four anhysteretic curve models considering four different materials, including isotropic, such as Mn-Zn soft ferrite, as well as anisotropic amorphous and nanocrystalline alloys. The presented results indicate that only the model that considers anisotropic energy is valid for a wide set of modern magnetic materials. The most suitable of the verified models is the anisotropic extension function-based model, which considers uniaxial anisotropy.

## 1. Introduction

The anhysteretic magnetization (AM) curve is one of the last problems posed by macroscopic models of magnetic hysteresis loops. It is extensively used in the modeling of soft magnetic materials. The AM curve is the basis of Jiles-Atherton model [1,2] and its modifications [3,4]. The Harrison model is also based on the AM curve [5]. The basic models of the AM curve itself, however, have not been validated experimentally for novel magnetic materials. As a result, this lack of validation of model usability presents a significant barrier for the development of advanced models of magnetic hysteresis loops, as well as the development of simplified models of the magnetization of soft magnetic materials. These simplified models are used during the design and optimization of soft magnetic devices during the optimization process on the basis of finite elements methods.

The anhysteretic magnetization curve can be measured experimentally by demagnetization of the magnetic material under the influence of a constant biasing magnetizing field [2]. However, the most obvious experimental method requires measurements of flux density in the sample during the demagnetization process [6]. Such measurements are very sophisticated from a technical point of view, and exhibit significant uncertainty due to various sources of drift of the integrators. As a result, despite the fact that the concept of the AM curve has been known of for over seventy years [7,8], a practical method for the measurement of such a curve for anisotropic materials was presented only recently [9].

Recent technological advances in reliable AM curve measurements has made it possible to fill the gap connected with the validation of known models of AM curves. This paper presents analyses of the accuracy of four recently used models of AM curve with respect to four modern soft magnetic materials. The presented results enable proper selection of an adequate AM curve model for isotropic or anisotropic materials. It also opens new possibilities for further analyses focused on understanding and quantitative description of the magnetization process in soft magnetic materials. This is especially important for the modeling of the newest classes of metastructures [10,11].

## 2. Models of Anhysteretic Curve

Due to the minimization of the total free energy of material [12], all ferromagnetic materials exhibit the domain structure [7]. As a result, the magnetization process is strongly influenced by inter-domain coupling quantified by Bloch interdomain coupling coefficient *α*. As a result, the efficient magnetization field *H_e_* in the ferromagnetic material is given by the following equation [13]:

(1) $$H_{e}=H+\alpha M$$

where *H* is the external magnetizing field and *M* is the magnetization of the material. 

Moreover, in all proposed models: *M_s_* is saturation magnetization [7], whereas parameter *a* determines the slope of the anhysteretic curve. It should also be considered that the flux density *B* in the material is given as:

(2) $$B=\left(M+H\right)\cdot \mu_{0}$$

where $\mu_{0}$ is the magnetic constant. 

The first model of anhysteretic magnetization curve utilizes erf() function (further called the “**erf-based model**”). This model is given by the following equation [14]:

(3) $$M\left(H\right)=M_{s}\cdot erf\left(\frac{H_{e}}{a}\right)$$

where erf() is given by the following equation:

(4) $$erf\left(x\right)=\frac{2}{\sqrt{\pi }}\int_{0}^{x}e^{-t^{2}}dt$$

The second model (further called the “**exp-based model**”) utilizes exponential dependence for the anhysteretic curve:

(5) $$M\left(H\right)=M_{s}\left(\frac{2}{1+e^{\frac{-1\cdot H}{a}}}-1\right)$$

The third model (further called the “**arctan function-based model**”) utilizes the arcus tangent function. This approach has previously been used for both amorphous [15] and nanomaterials [16]

(6) $$M\left(H\right)=M_{s}\frac{2}{\pi }\cdot arctan\left(\frac{H_{e}}{a}\right)$$

The fourth model (further called the “**Langevin function-based model**”) utilizes the Langevin equation, commonly used for modeling the magnetization curve of paramagnetic materials. Previously, it has commonly been used for modeling the anhysteretic curve of different materials in the Jiles-Atherton model [17,18,19,20]. The Langevin function-based model is given by the following equation [2]:

(7) $$M\left(H\right)=M_{s}\cdot \left(coth\left(\frac{H_{e}}{a}\right)-\frac{a}{H_{e}}\right)$$

which is determined by the Boltzman distribution of magnetic moments [21].

Similar assumptions lead to the last model (further called the “**anisotropic extension function-based model**”), which considers uniaxial anisotropy. The original model was presented by Ramesh et al. [22,23], and corrections have subsequently been proposed [6]:

(8) $$M\left(H\right)=M_{s}\left[\frac{\int_{0}^{\pi }e^{\frac{E\left(1\right)+E\left(2\right)}{2}}\sin\theta \cos\theta \cdot d\theta }{\int_{0}^{\pi }e^{\frac{E\left(1\right)+E\left(2\right)}{2}}\sin\theta \cdot d\theta }\right]$$

where for the uniaxial anisotropy [23]:

(9) $$E\left(1\right)=\frac{H_{e}}{a}\cos\theta -\frac{K_{an}}{\mu_{0}M_{s}a}\sin^{2}\left(\psi -\theta \right)$$

(10) $$E\left(2\right)=\frac{H_{e}}{a}\cos\theta -\frac{K_{an}}{\mu_{0}M_{s}a}\sin^{2}\left(\psi -\theta \right)$$

In these equations, *K_an_* represents average energy density of uniaxial anisotropy, whereas *ψ* is the angle between the easy axis of uniaxial anisotropy and direction of the magnetizing field *H*.

All of the presented mathematical models are initially normalized; however they include scaling factor *M_s_*_,_ which is actually a saturation magnetization of the material. In this way, the results of the modeling are in the same physical units as the measurement results.

## 3. Materials and Methods

There were four materials chosen for presented investigation: 

**Material 1:** Mn-Zn ferrite F3001, isotropic, with relatively high permeability (Polfer). Ring-shaped sample had outside diameter 40 mm, inside diameter 25 mm and height 18 mm, 

**Material 2:** Co_67_Fe_4_Mo_1_B_11_Si_17_ amorphous alloy, annealed, isotropic, with very high permeability (Amogreentech, Tongjin-eup, Korea). Ring-shaped sample had outside diameter 30 mm, inside diameter 20 mm and height 10 mm,

**Material 3:** Fe_73.5_Cu_1_Nb_3_Si_15.5_B_7_ nanocrystalline alloy with medium permeability and perpendicular anisotropy (Magnetec, NANOPERM LM, Langenselbold, Germany). Ring-shaped sample had outside diameter 30 mm, inside diameter 24 mm and height 6 mm,

**Materials 4:** Fe_67_Co_18_B_14_Si_1_ amorphous alloy, as cast, with very high permeability and parallel anisotropy (Metglas, 2605CO). Ring-shaped sample had outside diameter 32 mm, inside diameter 30 mm and height 10 mm.

Hysteresis loops with initial magnetization curves were measured with the hysteresisgraph system (Blacktower Ferrograph, ESP, Warsaw, Poland) [24]. Influence of external magnetic fields was compensated with Helmholtz coils, which is important for high-permeability materials. Samples were ring-shaped, with proper magnetizing and sensing windings. Other sample shapes are possible; however, they yield results of the sample properties, not the material properties, due to magnetic leakage, magnetizing force *H* uncertainty, and mainly the demagnetizing fields [7]. Amorphous alloys, due to their very high permeability, were magnetized with a single current-carrying rod. All of the ring-shaped cores were prepared by the listed producers, and are novel, commercially available inductive elements used in various technical applications.

**Figure 1:**
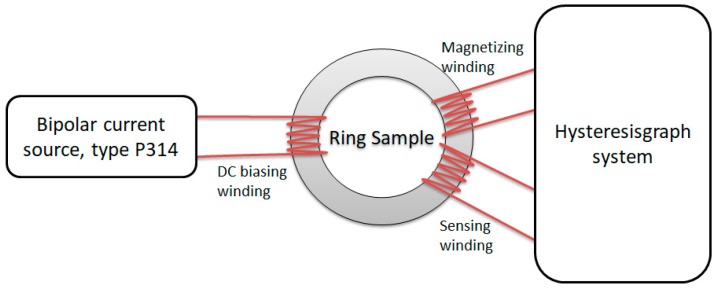
Schematic diagram of measurement test stand.

Anhysteretic magnetization curves were also measured with a Ferrograph system [24], using a slightly modified method published previously [9]. Details of the measurement procedures are fully described in [9]. The schematic diagram is presented in Figure 1. 

First, the samples were magnetized to saturation, and the hysteresis loops were recorded, giving an initial maximum induction *B*_max_*unbiased*_ value. Next, the *H_DC_* biasing field was incrementally added with the help of the third sample winding and P314 current-controlled bipolar power supply (Meratronik, Warsaw, Poland). In each step, the sample was demagnetized, and the biased hysteresis loop was measured, together with the initial magnetization curve, to obtain the maximum induction *B*_max__*_biased_* and the starting point of the initial magnetization curve $B_{0}$, which after DC biased demagnetization lies on the anhysteretic curve. The *H_AC_* magnetizing field amplitude of the Ferrograph system was incrementally decreased by the H_DC_ value to obtain a definite reference point on the *B*(*H*) diagram. The anhysteretic magnetization points (*H_a_*, *B_a_*) are thus given as:

(11) $$H_{a}=H_{DC},\;B_{a}=B_{\max\_unbiased}-\left(B_{\max\_biased}-B_{0}\right)$$

**Figure 2:**
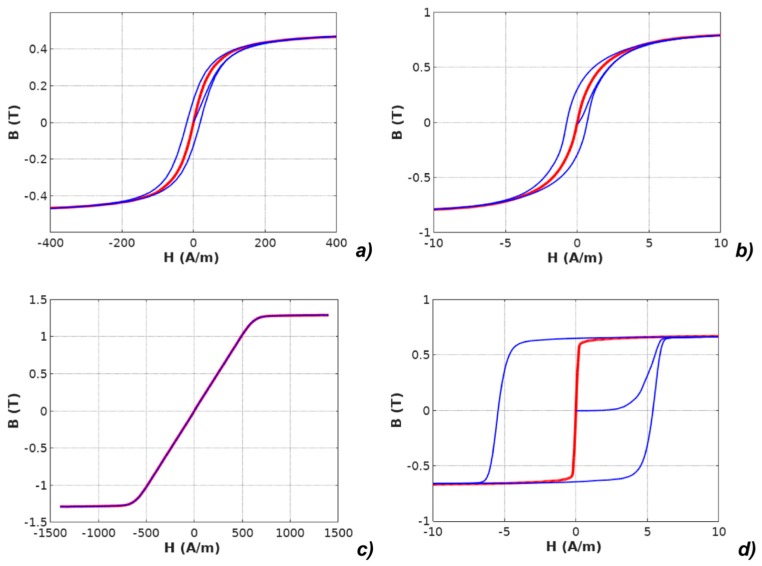
Results of measurements of hysteresis loops and anhysteretic magnetization curves (anhysteretic magnetization curve: red line, magnetic hysteresis loop: blue line): (**a**) Mn-Zn ferrite F3001, (**b**) Co_67_Fe_4_Mo_1_B_11_Si_17_ amorphous alloy, (**c**) Fe_73.5_Cu_1_Nb_3_Si_15.5_B_7_ nanocrystalline alloy with perpendicular anisotropy, (**d**) Fe_67_Co_18_B_14_Si_1_ amorphous alloy with parallel anisotropy.

The experimentally measured *B*(*H*) hysteresis loops and measured anhysteretic curves are presented in Figure 2.

## 4. Identification of Parameters of the Models

All four models of anhysteretic magnetization curves given by Equations (3)–(10) were implemented in Octave 4.4.1 (Free software, GNU Project, gnu.org/software/octave/), which is an open-source Matlab alternative. Identification of parameters for the models was performed by the optimization process utilizing a differential evolution algorithm. The target function *F* for optimization was given as a sum of squared differences between the results of experimental measurements of the anhysteretic curve and the results of its modeling:

(12) $$F=\overset{n}{\underset{i=1}{\sum }}{\left(B_{meas}\left(H_{i}\right)-B_{model}\left(H_{i}\right)\right)}^{2}$$

where *B_meas_(H_i_)* were the results of measurements for the set of given values of magnetizing field *H_i_*, whereas *B_model_(H_i_)* were the results of modeling for the same set of values of the magnetizing field *H_i_*. 

**Figure 3:**
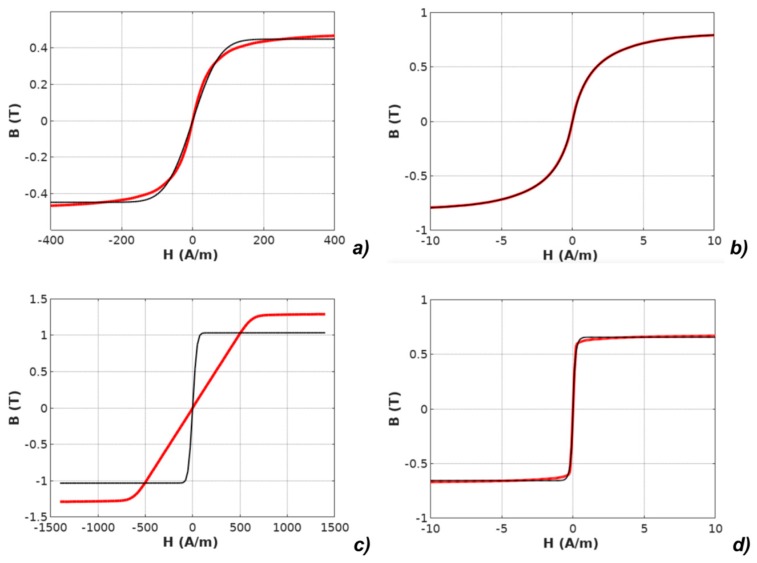
Results of modeling the anhysteretic curve using the erf-based function (measurements: red line, modeling: black line): (**a**) Mn-Zn ferrite F3001, (**b**) Co_67_Fe_4_Mo_1_B_11_Si_17_ amorphous alloy, (**c**) Fe_73.5_Cu_1_Nb_3_Si_15.5_B_7_ nanocrystalline alloy with perpendicular anisotropy, (**d**) Fe_67_Co_18_B_14_Si_1_ amorphous alloy with parallel anisotropy.

**Figure 4:**
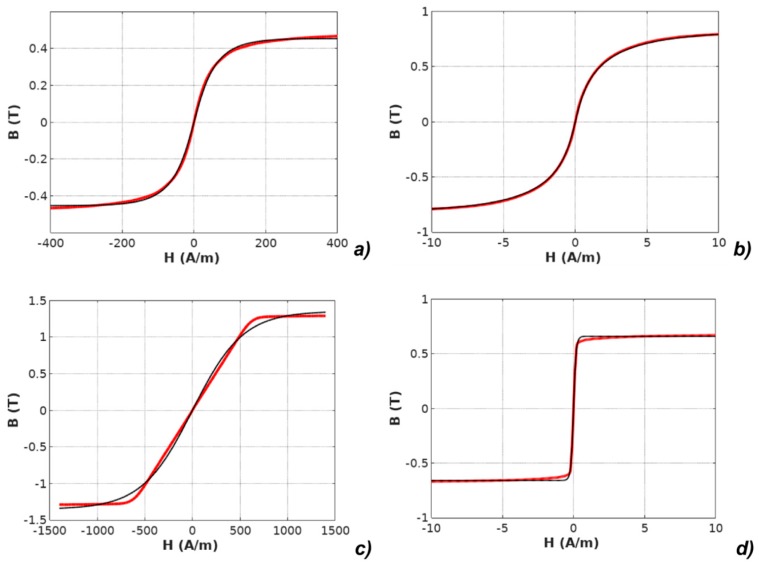
Results of modeling the anhysteretic curve using the exp-based function (measurements: red line, modeling: black line): (**a**) Mn-Zn ferrite F3001, (**b**) Co_67_Fe_4_Mo_1_B_11_Si_17_ amorphous alloy, (**c**) Fe_73.5_Cu_1_Nb_3_Si_15.5_B_7_ nanocrystalline alloy with perpendicular anisotropy, (**d**) Fe_67_Co_18_B_14_Si_1_ amorphous alloy with parallel anisotropy.

**Figure 5:**
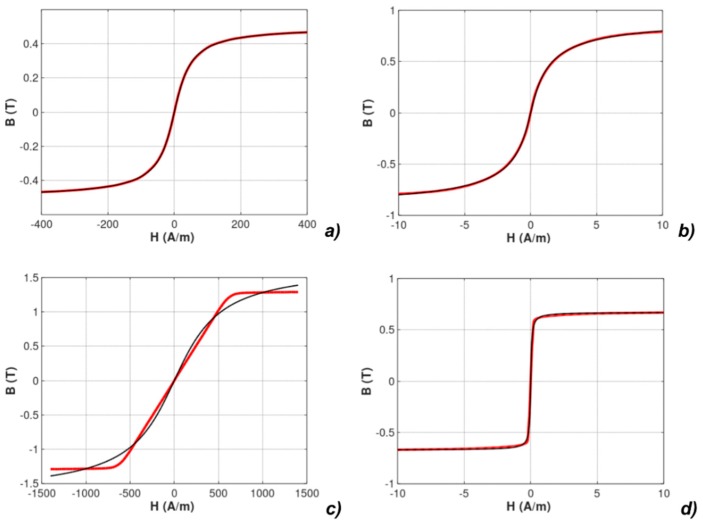
Results of modeling the anhysteretic curve using the arctan-based function (measurements: red line, modeling: black line): (**a**) Mn-Zn ferrite F3001, (**b**) Co_67_Fe_4_Mo_1_B_11_Si_17_ amorphous alloy, (**c**) Fe_73.5_Cu_1_Nb_3_Si_15.5_B_7_ nanocrystalline alloy with perpendicular anisotropy, (**d**) Fe_67_Co_18_B_14_Si_1_ amorphous alloy with parallel anisotropy.

**Figure 6:**
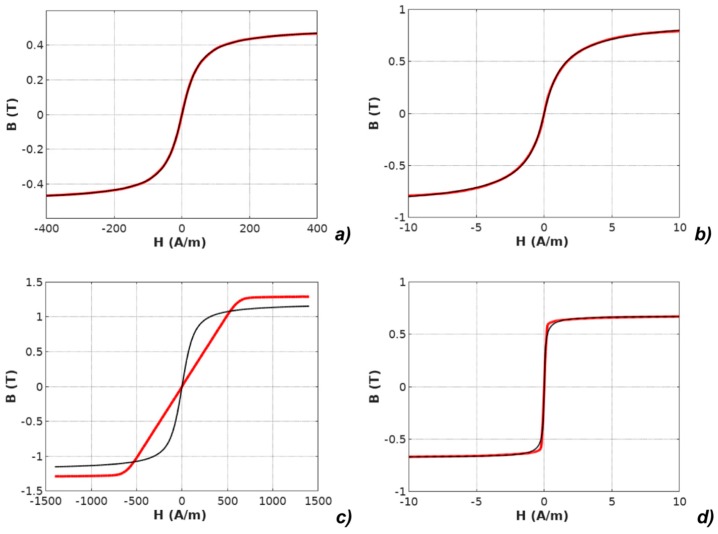
Results of modeling the anhysteretic curve using the isotropic Langevin function (measurements: red line, modeling: black line): (**a**) Mn-Zn ferrite F3001, (**b**) Co_67_Fe_4_Mo_1_B_11_Si_17_ amorphous alloy, (**c**) Fe_73.5_Cu_1_Nb_3_Si_15.5_B_7_ nanocrystalline alloy with perpendicular anisotropy, (**d**) Fe_67_Co_18_B_14_Si_1_ amorphous alloy with parallel anisotropy.

**Figure 7:**
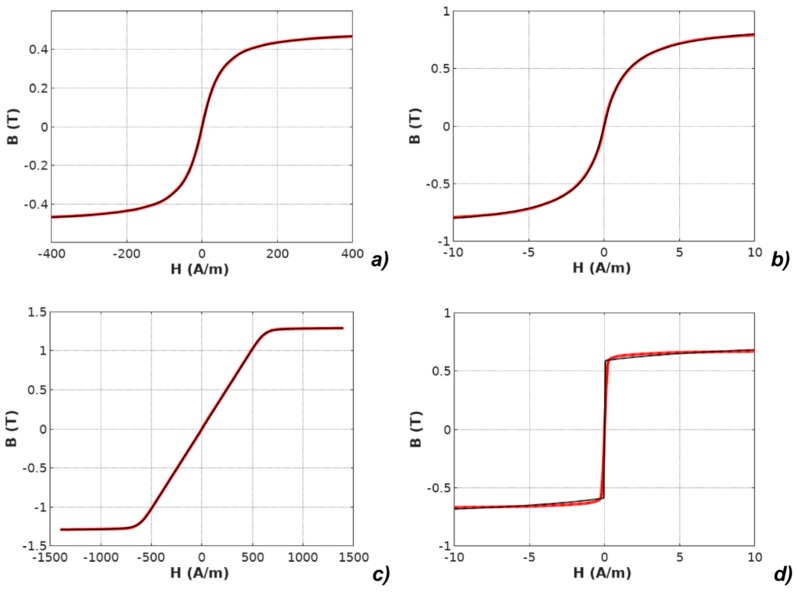
Results of modeling the anhysteretic curve using the anisotropic extension-based model (measurements: red line, modeling: black line): (**a**) Mn-Zn ferrite F3001, (**b**) Co_67_Fe_4_Mo_1_B_11_Si_17_ amorphous alloy, (**c**) Fe_73.5_Cu_1_Nb_3_Si_15.5_B_7_ nanocrystalline alloy with perpendicular anisotropy, (**d**) Fe_67_Co_18_B_14_Si_1_ amorphous alloy with parallel anisotropy.

It should be highlighted that the *M(H)* physical dependences described in the models were converted to *B*(*H*) dependences according to Equation (2). The results of modeling with the use of all four models applied for the results of measurements of all investigated materials are presented in Figure 3, Figure 4, Figure 5, Figure 6 and Figure 7.

The values of parameters determined during the optimization process are given in Table 1, Table 2, Table 3, Table 4 and Table 5. These tables also present the value of the R^2^ determination coefficient, which determines the part (in percent) of variance of the variable described by the model. As a result, R^2^ objectively quantifies the quality of the model.

The presented results clearly indicate that the accuracy of modeling strongly depends on the type of magnetic material. The erf-based model is suitable for isotropic amorphous materials, as well as for amorphous materials with parallel anisotropy. Both the exp-based model and the Langevin function-based model are only unsuitable for magnetic materials with strong perpendicular anisotropy. 

The anisotropic extension-based model proposed by Ramesh et al. [23] and corrected by Szewczyk [6] seems to be the most adequate for all types of magnetic materials. It is able to properly represent the anhysteretic magnetization of isotropic materials (such as soft Mn-Zn ferrites), as well as anisotropic materials (like amorphous alloys with different types of anisotropy). Flexibility and adequacy of this model is connected with its physical background, utilizing assumption of Boltzman distribution of domains magnetization directions in ferromagnetic material. On the other hand, the anisotropic extension-based model is the most sophisticated among presented models and consumes the most computing resources. However, this drawback is less significant due to the constant increase of computing power of modern computer systems used for modeling.

A general assessment of the models’ accuracy is presented in Table 6. This table may be a guideline for researchers and engineers searching for the appropriate model for anhysteretic magnetization for the modeling of devices with inductive cores made of different types of soft magnetic materials.

## 5. Conclusions

Novel advances in the area of experimental measurements of anhysteretic magnetization curve create new possibilities in the validation of models of magnetization process. This validation will enable adequate selection of anhysteretic magnetization curve model for both modeling of magnetization process and hysteresis loops as well as for engineering applications.

The results presented in this paper indicate that not all commonly used models of anhysteretic magnetization curves are suitable for all types of soft magnetic materials. Especially for amorphous alloys with strong perpendicular anisotropy, only the anisotropic extension-based model enables modeling with an accuracy described by *R*^2^ coefficient exceeding 99.999%. On the other hand, the commonly used Langevin-function-based model is fully adequate for isotropic materials, such as soft Mn-Zn ferrites. For the generalized case, most of the commercially available soft magnetic materials fall into one of these three categories: isotropic, parallel anisotropic, or perpendicular anisotropic. The presented models are thus expected to work with similar to presented accuracy. The limitations of the presented work are novel, experimental cases of two-phase materials, or materials with cubic anisotropy—but these are still uncommon, and, to the authors’ knowledge, for these materials, no suitable AM curve model has been presented.

The experimental results clearly indicate that physical principles-based models of AM curve are most accurate for modeling the characteristics of modern magnetic materials. This fact should be taken into consideration by both researchers trying to understand the magnetic hysteresis mechanisms, as well as by engineers developing components with cores made of soft magnetic materials, such as magnetic field sensors [25] and novel magnetic circuits [26]. Moreover, the results of magnetic measurements confirm the results of previous research concerning the Cu addition to ferromagnetic alloys [27].

## Figures and Tables

**Table 1:** Parameters of anhysteretic curve erf-based model identified during the optimization process.

Parameter	Unit	Mn-Zn ferrite F3001	Co_67_Fe_4_Mo_1_B_11_Si_17_	Fe_73.5_Cu_1_Nb_3_Si_15.5_B_7_ Perpendicular Anisotropy	Fe_67_Co_18_B_14_Si_1_ Parallel Anisotropy
*M_s_*	A/m	356,460	643,597	820,551	521,407
*a*	A/m	87.52	11.20	50.00	0.52
*α*		7.65 × 10^−7^	1.31 × 10^−5^	2.92 × 10^−8^	5.96 × 10^−7^
*R* ^2^	%	99.79	99.9989	88.47	99.96

**Table 2:** Parameters of anhysteretic curve exp-based model identified during the optimization process.

Parameter	Unit	Mn-Zn ferrite F3001	Co_67_Fe_4_Mo_1_B_11_Si_17_	Fe_73.5_Cu_1_Nb_3_Si_15.5_B_7_ Perpendicular Anisotropy	Fe_67_Co_18_B_14_Si_1_ Parallel Anisotropy
*M_s_*	A/m	360,958	649,693	1,073,424	523,969
*a*	A/m	55.90	3.99	264.94	0.10
*α*		1.34 × 10^−5^	9.91 × 10^−6^	2.92 × 10^−8^	5.64 × 10^−8^
*R* ^2^	%	99.94	99.9977	99.74	99.96

**Table 3:** Parameters of anhysteretic curve atan-based model identified during the optimization process.

Parameter	Unit	Mn-Zn ferrite F3001	Co_67_Fe_4_Mo_1_B_11_Si_17_	Fe_73.5_Cu_1_Nb_3_Si_15.5_B_7_ Perpendicular Anisotropy	Fe_67_Co_18_B_14_Si_1_ Parallel Anisotropy
*M_s_*	A/m	399,291	727,548	1,330,538	533,825
*a*	A/m	44.01	2.36	385.76	0.08
*α*		2.26 × 10^−5^	2.68 × 10^−6^	2.94 × 10^−8^	5.64 × 10^−8^
*R* ^2^	%	99.9992	99.995	99.50	99.97

**Table 4:** Parameters of anhysteretic curve Langevin-based model identified during the optimization process.

Parameter	Unit	Mn-Zn ferrite	Co_67_Fe_4_Mo_1_B_11_Si_17_	Fe_73.5_Cu_1_Nb_3_Si_15.5_B_7_ Perpendicular Anisotropy	Fe_67_Co_18_B_14_Si_1_ Parallel Anisotropy
*M_s_*	A/m	402,878	737,851	948,825	537,164
*a*	A/m	32.67	1.84	50.00	0.10
*α*		9.17 × 10^−5^	5.07 × 10^−5^	2.92 × 10^−8^	3.68 × 10^−7^
*R* ^2^	%	99.9990	99.995	95.11	99.95

**Table 5:** Parameters of anhysteretic curve with anisotropic extension-based model identified during the optimization process.

Parameter	Unit	Mn-Zn ferrite F-3001	Co_67_Fe_4_Mo_1_B_11_Si_17_	Fe_73.5_Cu_1_Nb_3_Si_15.5_B_7_ Perpendicular Anisotropy	Fe_67_Co_18_B_14_Si_1_ Parallel Anisotropy
*M_s_*	A/m	403,075	736,367	1,028,169	602,000
*a*	A/m	32.98	1.80	2.72	28.86
*α*		9.5 × 10^−5^	4.8 × 10^−5^	4.48 × 10^−6^	6.85 × 10^−5^
*Κ_an_*	J/m^3^	0.05	0.04	411.42	487.98
*R* ^2^	%	99.9990	99.995	99.9993	99.65

where: *M_s_*—saturation magnetization, *a*—anhysteretic curve slope coefficient, *α*—Bloch interdomain coupling coefficient, *Κ_an_*—average energy density of uniaxial anisotropy coefficient, *R*^2^—coefficient of determination.

**Table 6:** General assessment of model’s accuracy quantified by *R*^2^ parameter expressed in percentages (Green—good, orange—poor, red—very poor).

*R*^2^ (%)	Mn-Zn ferrite F-3001	Co_67_Fe_4_Mo_1_B_11_Si_17_	Fe_73.5_Cu_1_Nb_3_Si_15.5_B_7_ Perpendicular Anisotropy	Fe_67_Co_18_B_14_Si_1_ Parallel Anisotropy
**erf-based**	99.79	**99.9989**	88.47	99.96
**exp-based**	99.94	**99.9977**	99.74	99.96
**arctan-based**	**99.9992**	**99.995**	99.50	99.97
**Langevin function-based**	**99.9990**	**99.995**	95.11	99.95
**Anisotropic extension-based**	**99.9990**	**99.995**	**99.9993**	99.65
